# Case Report: Trousseau syndrome presenting as multifocal cerebral infarctions in advanced gastric signet-ring cell carcinoma: tumor burden–driven hypercoagulability

**DOI:** 10.3389/fonc.2026.1818823

**Published:** 2026-07-15

**Authors:** Haocheng Zhao, Shanshan Lin, Wenye Huang

**Affiliations:** 1 Department of Hematology, Yueqing People’s Hospital, Yueqing, Zhejiang, China; 2 Department of Respiratory Medicine, Yueqing People’s Hospital, Yueqing, Zhejiang, China

**Keywords:** chemotherapy, D-dimer, gastric signet-ring cell carcinoma, malignancy-associated hypercoagulability, Trousseau syndrome, tumor burden

## Abstract

**Background:**

Malignancy-associated hypercoagulability is a well-recognized driver of thromboembolic events in patients with advanced cancer. Arterial complications, including ischemic stroke, can develop despite appropriate anticoagulation and are often accompanied by markedly elevated D-dimer levels, reflecting sustained coagulation activation in the setting of advanced malignancy.

**Case presentation:**

A 76-year-old man presented with gastrointestinal bleeding and was subsequently diagnosed with advanced gastric signet-ring cell carcinoma with extensive hepatic and retroperitoneal metastases. During hospitalization, he developed acute left-sided hemiplegia. Brain magnetic resonance imaging revealed multiple acute infarctions involving both cerebral hemispheres and the cerebellum across distinct vascular territories, consistent with a systemic embolic pattern. The D-dimer level increased to approximately 23.7 μg/mL at the time of neurological deterioration. After gastrointestinal bleeding had stabilized, anticoagulation with enoxaparin sodium 4,000 anti-Xa IU subcutaneously twice daily was initiated; however, D-dimer remained elevated at approximately 23.1 μg/mL on May 21. Following initiation of systemic chemotherapy with the XELOX regimen, neurological deficits improved, accompanied by a decline in D-dimer from approximately 17.5 μg/mL before chemotherapy to approximately 14.9 μg/mL on June 2.

**Conclusion:**

This case underscores the characteristic radiological features of malignancy-associated stroke and highlights the close relationship between tumor burden and systemic hypercoagulability. The observed temporal association between chemotherapy initiation and reduction in D-dimer levels supports the concept that effective tumor-directed therapy, in addition to anticoagulation, may be necessary to mitigate cancer-related thrombotic complications. Early multidisciplinary intervention is essential to optimize both neurological recovery and oncologic management.

## 1 Introduction

Cancer-associated thrombosis is a leading cause of morbidity and mortality in patients with malignancy (1). Although venous thromboembolism remains the most recognized manifestation, arterial thrombotic events—particularly ischemic stroke—are increasingly appreciated as clinically consequential complications of cancer-related hypercoagulability (2, 3). Malignancy-associated stroke frequently presents with multifocal infarctions involving multiple vascular territories and is often accompanied by markedly elevated D-dimer levels, reflecting ongoing activation of coagulation and fibrinolytic pathways (4–6).

Among solid tumors, mucin-producing adenocarcinomas, especially those of gastrointestinal origin, carry a particularly high thromboembolic risk (7, 8). Gastric signet-ring cell carcinoma, characterized by abundant intracellular mucin and diffuse infiltrative growth, represents a histologic subtype with notable prothrombotic potential (9, 10). Several mechanisms have been proposed, including tumor-derived tissue factor expression, cancer procoagulant activity, mucin-mediated platelet aggregation through selectin interactions, and amplification of systemic inflammatory responses (9, 11, 12). Despite growing insight into these mechanisms, management of malignancy-associated hypercoagulability remains complex—particularly when thrombotic events develop despite appropriate anticoagulation (13, 14).

The relationship between tumor burden, circulating coagulation markers, and neurological outcomes in individual patients remains incompletely defined (3, 4). In particular, it is unclear to what extent effective tumor-directed therapy can reverse hypercoagulability and alter the clinical course of cancer-associated stroke (14, 15).

We describe a patient with advanced gastric signet-ring cell carcinoma who developed multifocal cerebral infarctions consistent with malignancy-associated hypercoagulability (16, 17). This case emphasizes the characteristic imaging features of cancer-associated stroke, the persistence of elevated D-dimer levels despite anticoagulation, and the subsequent clinical and biochemical changes observed after initiation of systemic chemotherapy (4, 5).

## 2 Case presentation

A 76-year-old man was admitted on May 7, 2025, with acute dizziness accompanied by nausea, vomiting, and melena. He had no history of hypertension, diabetes mellitus, atrial fibrillation, coronary heart disease, myocardial infarction, valvular heart disease, heart failure, previous ischemic stroke, transient ischemic attack, or intracerebral hemorrhage, and he was a non-smoker. Initial laboratory evaluation revealed severe anemia, with a hemoglobin level of 73 g/L (reference range, 130–175 g/L). Emergency cranial computed tomography showed no acute intracranial abnormalities. However, contrast-enhanced chest and abdominal computed tomography incidentally identified a mass in the left hepatic lobe, raising concern for an underlying malignancy.

Subsequent oncologic workup revealed markedly elevated tumor markers, including CA19-9 (>12,000 U/mL; reference range, 0–37 U/mL), CA125 (416.7 U/mL; reference range, 0–35 U/mL), CA72-4 (156.00 U/mL; reference range, 0–6.9 U/mL), and carcinoembryonic antigen (17.3 ng/mL; reference range, 0–5 ng/mL), while alpha-fetoprotein remained within normal limits (4.90 ng/mL; reference range, 0–7 ng/mL). Coagulation testing demonstrated a substantially elevated D-dimer level of 18.45 μg/mL(reference range, 0.00–0.55 μg/mL). D-dimer was measured by immunoturbidimetry using original Mindray reagents on a Mindray CX9000 automated coagulation analyzer and reported in μg/mL, with an institutional threshold of 0.55 μg/mL used for VTE exclusion. The platelet count was 165 × 10^9/L on May 8 (institutional reference interval, 125–350 × 10^9/L). At the time of acute neurological deterioration on May 16, the platelet count was 121 × 10^9/L. Fecal occult blood testing was positive. After stabilization of gastrointestinal bleeding, contrast-enhanced abdominal magnetic resonance imaging was obtained, revealing a large heterogeneous lesion in the left hepatic lobe, accompanied by multiple intrahepatic nodules and retroperitoneal lymphadenopathy—findings consistent with extensive metastatic disease (**Figure 1**).

**Figure 1:**
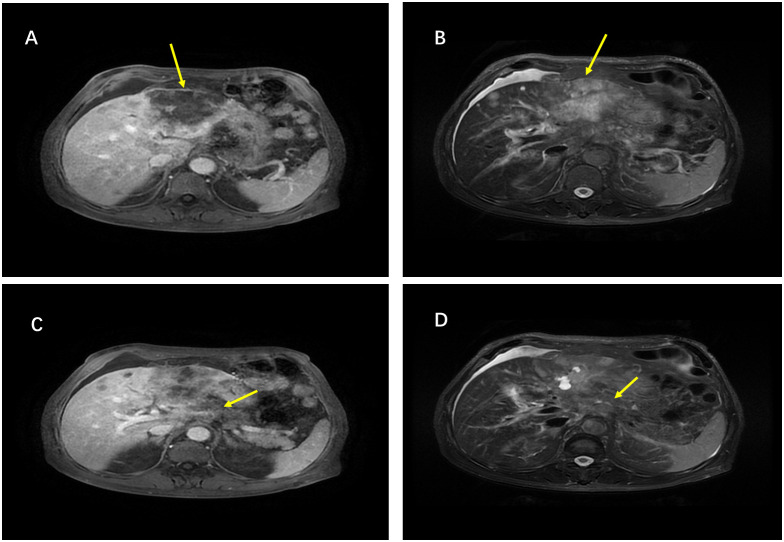
Contrast-enhanced abdominal magnetic resonance imaging demonstrating extensive hepatic and retroperitoneal tumor involvement. Axial contrast-enhanced T1-weighted imaging **(A, C)** and fat-suppressed T2-weighted imaging **(B, D)** reveal a large heterogeneously enhancing mass in the left hepatic lobe (approximately 5–8 cm in maximal diameter; yellow arrow) with irregular margins. Multiple additional intrahepatic nodular lesions are present throughout both lobes, appearing hyperintense on T2-weighted images and enhancing on post-contrast sequences, consistent with diffuse intrahepatic tumor involvement. Enlarged enhancing lymph nodes are identified along the para-aortic retroperitoneal region (yellow arrows), appearing hyperintense on T2-weighted images with well-defined borders.

Because the imaging findings were not entirely explained by the tumor marker profile, upper gastrointestinal endoscopy was performed. An ulcerative lesion was observed in the pyloric canal (**Figure 2A**). Histopathological analysis of biopsy specimens confirmed signet-ring cell carcinoma (**Figures 2B, C**). The patient was therefore diagnosed with advanced gastric signet-ring cell carcinoma with hepatic and retroperitoneal metastases.

**Figure 2:**
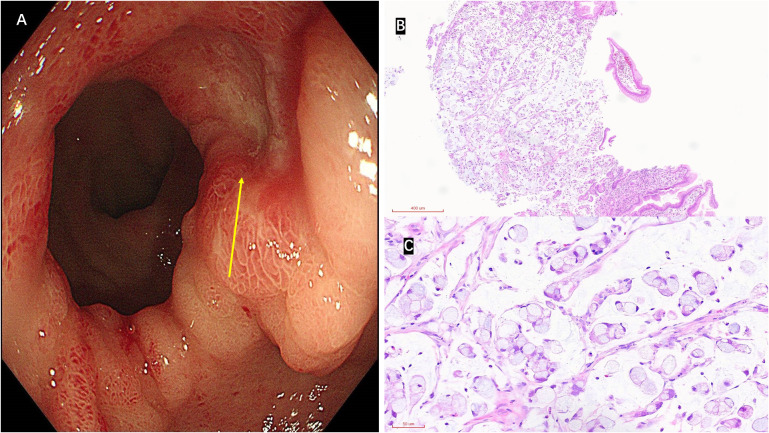
Endoscopic and histopathological features of gastric signet-ring cell carcinoma. Upper gastrointestinal endoscopy **(A)** demonstrates an ulcerative lesion located in the pyloric canal, characterized by a central depression and adherent whitish exudate (yellow arrow). The surrounding mucosa appears erythematous and edematous. Hematoxylin and eosin (H&E) staining at low magnification **(B)** shows diffuse infiltration of tumor cells within the gastric mucosa, accompanied by abundant extracellular mucin. High-power examination **(C)** reveals numerous signet-ring cells with prominent intracytoplasmic mucin displacing the nuclei toward the periphery, consistent with the characteristic morphology of signet-ring cell carcinoma.

On May 16, the patient developed acute confusion and sudden left-sided hemiplegia. Neurological examination demonstrated complete motor paralysis (Medical Research Council grade 0) of the left upper and lower extremities. Brain magnetic resonance imaging revealed multiple acute infarctions involving both cerebral hemispheres and the cerebellum across distinct vascular territories (**Figure 3**). Electrocardiography showed sinus rhythm with occasional premature atrial contractions, without evidence of atrial fibrillation. Emergency transthoracic echocardiography showed preserved left ventricular systolic function, with an ejection fraction of approximately 67%, and no obvious intracardiac structural abnormalities, valvular abnormalities, or abnormal Doppler flow. At that time, the D-dimer level had further increased to 23.65 μg/mL. In the setting of advanced malignancy and multifocal embolic infarctions, malignancy-associated hypercoagulability (Trousseau syndrome) was considered the most likely diagnosis.

**Figure 3:**
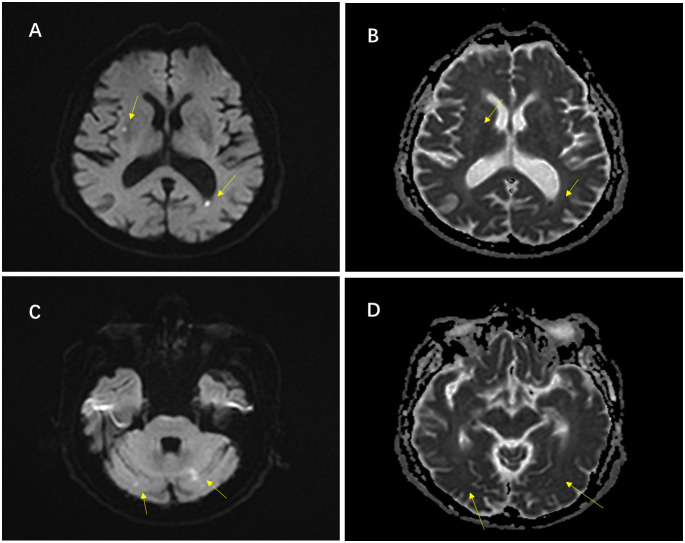
Brain magnetic resonance imaging demonstrating multifocal acute infarctions across multiple vascular territories. Axial diffusion-weighted imaging (DWI) **(A, C)** shows multiple scattered hyperintense lesions involving bilateral cortical and subcortical regions (yellow arrows), distributed across distinct vascular territories. Additional DWI images demonstrate hyperintense lesions within the cerebellar hemispheres (yellow arrows), indicating posterior circulation involvement. Corresponding apparent diffusion coefficient (ADC) maps **(B, D)** reveal reduced signal intensity in the same regions, confirming diffusion restriction consistent with acute infarction.

Following multidisciplinary consultation, therapeutic anticoagulation with enoxaparin (0.4 mL subcutaneously twice daily) was initiated after gastrointestinal bleeding had stabilized. Despite appropriate anticoagulation, D-dimer levels remained persistently elevated, and neurological recovery was limited. The patient subsequently experienced a generalized seizure, suggesting ongoing thromboembolic activity.

Given the inadequate response to anticoagulation alone, systemic chemotherapy with the XELOX regimen (oxaliplatin plus capecitabine) was initiated on May 31. Within three days of treatment initiation, neurological function improved, with left-sided muscle strength recovering from Medical Research Council grade 0 to grade 3. Concurrently, D-dimer levels decreased from 17.48 μg/mL prior to chemotherapy to 14.94 μg/mL on June 2 and remained lower on subsequent measurements (**Figure 4**).

**Figure 4:**
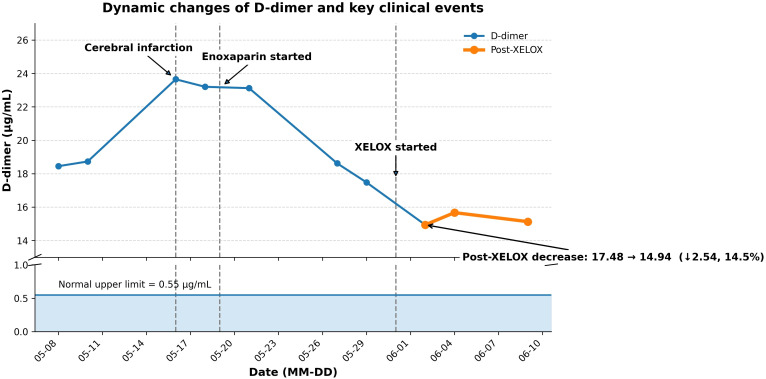
Temporal changes in plasma D-dimer levels during hospitalization. Serial plasma D-dimer measurements are shown over the course of hospitalization. A marked elevation was observed at the time of acute cerebral infarction (yellow arrow). Therapeutic anticoagulation with enoxaparin was subsequently initiated; however, D-dimer levels remained elevated. Following initiation of systemic chemotherapy with the XELOX regimen (yellow arrow), D-dimer levels decreased from 17.48 to 14.94 μg/mL. The shaded area represents the upper limit of the normal reference range (≤0.55 μg/mL).

The patient’s condition stabilized, and he was discharged on July 1, 2025. A detailed timeline of clinical events and dynamic laboratory changes is provided in **Table 1**.

**Table 1:** Timeline of major clinical events and dynamic changes in coagulation markers during hospitalization.

Date	Clinical events and key findings	D-dimer (μg/mL)	Fibrinogen (g/L)	Management
May 7, 2025	Admission for dizziness, nausea, vomiting, and melena; Hb 73 g/L; CT revealed a left hepatic mass	—	—	Supportive treatment for gastrointestinal bleeding
May 8	Initial oncologic and coagulation workup; CA19-9 >12,000 U/mL; markedly elevated D-dimer	18.45	0.86	—
May 12	Contrast-enhanced abdominal MRI showed a large left hepatic lesion, multiple intrahepatic metastatic nodules, and retroperitoneal lymphadenopathy	—	—	—
May 13	Upper gastrointestinal endoscopy revealed an ulcerative lesion in the pyloric canal	—	—	Endoscopic biopsy
May 15	Histopathology confirmed gastric signet-ring cell carcinoma	—	—	Diagnosis of metastatic gastric carcinoma
May 16	Acute neurological deterioration with sudden left-sided hemiplegia	23.65	0.74	—
May 17	Brain MRI showed multiple acute infarctions involving bilateral cerebral hemispheres and the cerebellum	—	—	Antiplatelet therapy initiated
May 18-19	Persistent hypercoagulability; multidisciplinary evaluation established the diagnosis of Trousseau syndrome	23.20	1.00	Enoxaparin initiated (0.4 mL q12h)
May 21	No significant neurological improvement after anticoagulation	23.12	1.54	Continued anticoagulation
May 29	Seizure episode with clinical deterioration	17.48	2.93	Supportive management
May 31	Initiation of systemic chemotherapy with the XELOX regimen	—	—	Oxaliplatin + capecitabine
June 2	Early post-chemotherapy reassessment showed improved mental status and recovery of left-sided muscle strength from grade 0 to grade 3	14.94	2.21	Continued chemotherapy
June 9	Follow-up evaluation showed clinical stabilization	15.13	2.37	—
July 1	Discharge after clinical stabilization; family declined further antitumor therapy	—	—	—

D-dimer values are reported in μg/mL. D-dimer was measured by immunoturbidimetry using original Mindray reagents on a Mindray CX9000 automated coagulation analyzer, and the institutional threshold used for VTE exclusion was 0.55 μg/mL. The institutional reference interval for fibrinogen was 2.00–4.00 g/L.

## 3 Discussion

Malignancy-associated hypercoagulability is a complex paraneoplastic process in which tumor biology actively drives systemic thrombosis (1, 11). Among solid tumors, mucin-producing adenocarcinomas—particularly those arising from the gastrointestinal tract—are consistently associated with an increased thromboembolic risk (7, 8). Gastric signet-ring cell carcinoma, defined by abundant intracellular mucin and diffuse infiltrative growth, represents a histologic subtype with notable prothrombotic potential (9, 10).

The neuroimaging findings in this patient—multiple acute infarctions involving both cerebral hemispheres and the posterior circulation (**Figure 3**)—strongly support a systemic embolic mechanism rather than large-vessel atherosclerotic disease (3, 4). Dedicated cerebrovascular imaging, such as computed tomography angiography or magnetic resonance angiography, was not performed because of the patient’s poor performance status, recent gastrointestinal bleeding, and advanced metastatic disease. Therefore, the absence of cerebral vascular stenosis could not be directly confirmed, which represents a limitation of this case. Nevertheless, the multifocal infarctions involving bilateral anterior and posterior circulation territories, together with markedly elevated D-dimer levels and advanced malignancy, were more consistent with cancer-associated hypercoagulability than with a single large-artery stenotic mechanism. Multifocal infarctions affecting distinct vascular territories are widely recognized as a characteristic pattern of cancer-associated stroke (3, 4, 6). This so-called “embolic shower” distribution reflects circulating procoagulant activity and microthrombus formation, rather than focal arterial pathology, and should raise suspicion for an underlying malignancy in the appropriate clinical context (2, 18).

The mechanisms underlying malignancy-associated hypercoagulability are multifaceted. Tumor cells may express tissue factor, initiating the extrinsic coagulation cascade and promoting thrombin generation (11, 12). Cancer procoagulant, a cysteine protease selectively expressed by malignant cells, can further activate factor X independently of tissue factor (11, 12). In mucin-rich tumors, circulating mucins interact with P- and L-selectins on platelets, leukocytes, and endothelial cells, facilitating platelet aggregation and microvascular thrombosis (9). Increasing evidence supports the central role of these selectin-mediated interactions in mucin-associated thrombosis, offering a biological explanation for the heightened thrombotic propensity observed in gastrointestinal signet-ring carcinomas (10, 12).

In this case, the extent of hepatic metastases and retroperitoneal lymphadenopathy (**Figure 1**) reflected a substantial tumor burden (1, 3). A high tumor burden may sustain systemic coagulation activation through continuous release of tissue factor–bearing microparticles, mucins, and proinflammatory cytokines (1, 11, 12). Suspected portal venous involvement on imaging may have further compounded both local hemodynamic disturbance and systemic procoagulant signaling (8). The markedly elevated CA19–9 level likely reflected both tumor volume and mucin production, and may indirectly mirror the degree of hypercoagulable activity (7, 9).

A notable clinical feature in this patient was the limited response to anticoagulation alone. Despite therapeutic low–molecular-weight heparin, D-dimer levels remained elevated and neurological deficits progressed (3, 14). This observation aligns with prior reports indicating that anticoagulation can reduce thrombus propagation but does not address the underlying tumor-driven stimulus sustaining the hypercoagulable state (13, 14). As long as the malignancy remains biologically active, coagulation activation may persist despite adequate antithrombotic therapy (1, 14).

Following initiation of systemic chemotherapy, however, both neurological status and D-dimer levels improved (3, 15). Although causality cannot be definitively established in a single case, the temporal relationship between chemotherapy and reduction in D-dimer supports the possibility that effective tumor control attenuated the procoagulant milieu (5, 15). A reduction in viable tumor mass may decrease circulating prothrombotic mediators and inflammatory signaling, thereby dampening systemic coagulation activation (1, 11).

This case also underscores the potential utility of D-dimer beyond its conventional diagnostic role (5). In addition to D-dimer, fibrinogen is an important coagulation-related biomarker in patients with malignancy, and abnormalities in these markers have been associated with adverse oncologic outcomes. In this setting, D-dimer appeared to function as a dynamic indicator of tumor-driven coagulation activity (4, 5). The parallel improvement in neurological function and decline in D-dimer levels suggests that coagulation markers may reflect ongoing tumor–host interactions in real time (3, 5). Serial monitoring of D-dimer may therefore provide clinically relevant insight into both thrombotic risk and response to oncologic therapy in selected patients with cancer-associated stroke (4, 5).

From a management standpoint, these observations reinforce the importance of a dual therapeutic approach: anticoagulation to limit further thrombus formation and timely tumor-directed therapy to reduce the biological drivers of hypercoagulability (13, 14). Close collaboration among oncology, neurology, and hematology teams is particularly critical when thrombotic events arise despite appropriate anticoagulation (1, 14).

Overall, this case integrates imaging, laboratory, and therapeutic data to support a tumor-driven mechanism of hypercoagulability in advanced gastric signet-ring cell carcinoma (4, 9). It highlights the dynamic relationship between tumor burden and systemic coagulation activity and suggests that effective oncologic treatment may be pivotal in reversing cancer-associated thrombotic complications (3, 14).

## 4 Conclusions

This case highlights the importance of maintaining a high index of suspicion for malignancy-associated hypercoagulability in patients with advanced cancer who present with multifocal ischemic stroke and markedly elevated D-dimer levels. Recognition of infarctions involving multiple vascular territories should prompt consideration of an underlying tumor-driven prothrombotic state.

The clinical course observed here suggests that anticoagulation alone may be insufficient when hypercoagulability is sustained by active tumor burden. In such circumstances, timely initiation of tumor-directed therapy, in conjunction with appropriate antithrombotic treatment, may be necessary. Close collaboration among oncology, neurology, and hematology teams is essential to optimize both neurological and oncologic outcomes.

## Data Availability

The original contributions presented in the study are included in the article/supplementary material. Further inquiries can be directed to the corresponding author.
